# Top-Cited Articles in Traumatic Brain Injury

**DOI:** 10.3389/fnhum.2014.00879

**Published:** 2014-11-05

**Authors:** Bhanu Sharma, David Wyndham Lawrence

**Affiliations:** ^1^Toronto Rehabilitation Institute, University of Toronto, Toronto, ON, Canada; ^2^Department of Family and Community Medicine, St. Michael’s Hospital, University of Toronto, Toronto, ON, Canada

**Keywords:** traumatic brain injury, head injury, concussion, bibliometric, citation

## Abstract

A review of the top-cited articles in a scientific discipline can identify areas of research that are well established and those in need of further development, and may, as a result, inform and direct future research efforts. Our objective was to identify and characterize the top-cited articles in traumatic brain injury (TBI). We used publically available software to identify the 50 TBI articles with the most lifetime citations, and the 50 TBI articles with the highest annual citation rates. A total of 73 articles were included in this review, with 27 of the 50 papers with the highest annual citation rates common to the cohort of 50 articles with the most lifetime citations. All papers were categorized by their primary topic or focus, namely: predictor of outcome, pathology/natural history, treatment, guidelines and consensus statements, epidemiology, assessment measures, or experimental model of TBI. The mean year of publication of the articles with the most lifetime citations and highest annual citation rates was 1990 ± 14.9 years and 2003 ± 6.7 years, respectively. The 50 articles with the most lifetime citations typically studied predictors of outcome (34.0%, 17/50) and were specific to severe TBI (38.0%, 19/50). In contrast, the most common subject of papers with the highest annual citation rates was treatment of brain injury (22.0%, 11/50), and these papers most frequently investigated mild TBI (36.0%, 18/50). These findings suggest an intensified focus on mild TBI, which is perhaps a response to the dedicated attention these injuries are currently receiving in the context of sports and war, and because of their increasing incidence in developing nations. Our findings also indicate increased focus on treatment of TBI, possibly due to the limited efficacy of current interventions for brain injury. This review provides a cross-sectional summary of some of the most influential articles in TBI, and a bibliometric examination of the current status of TBI research.

## Introduction

In an effort to improve clinical outcomes following traumatic brain injury (TBI), leading scientific institutions have pioneered an international, multidisciplinary research initiative (The Lancet, 2012). In particular, the Canadian Institutes of Health Research, the National Institutes of Health, and the European Commission – leading research agencies within their respective jurisdictions – have recently collaborated to fund and advance TBI research through the International Initiative for Traumatic Brain Injury Research (European Commission, 2012; The Lancet, 2012; The Lancet Neurology, 2013; Tosetti et al., 2013). This unprecedented effort at TBI-specific international collaboration and resource pooling is a testament to the global burden of this injury (The Lancet Neurology, 2013), and it is perhaps a response to calls for increased funding for brain injury research and greater networking between institutions studying TBI (Zitnay et al., 2008). The pressing need to advance TBI research is most evident when considering predictions that TBI will become the third leading cause of death and disability *worldwide* by the year 2020 (World Heath Organization, 2002; The Lancet, 2012).

Commitments to advance our understanding of TBI and the management strategies available to treat this injury will be effectuated by an increase in research activity (The Lancet, 2012). It is important, therefore, to examine current TBI literature and identify the areas of research that are well established and those in need of further development, as this may inform researchers and granting agencies where to focus future research efforts. Reviewing the literature with this aim is especially important considering that consensus statements report that our understanding of TBI has progressed further in some areas than in others (Zitnay et al., 2008).

One way to objectively identify a well-developed area of the literature is to measure the number of citations it has accumulated (Patsopoulos et al., 2005; Ponce and Lozano, 2010). Publications that are highly cited are well recognized, widely read and referenced, regularly discussed, and likely to be considered important within their respective subfields (Lipsman et al., 2014). Moreover, the number of times a publication is cited serves as a proxy for its influence within a discipline (Garfield, 1986; Lipsman et al., 2014). An analysis of the top-cited articles in a given field, therefore, provides clinicians and researchers with a cross-sectional summary of some of the most important work on a topic (Yang and Pan, 2006; Ibrahim et al., 2012; Lipsman and Lozano, 2012; Lipsman et al., 2014). Bibliometric citation analyses can also reveal the breadth (Lipsman et al., 2014) and existing patterns or themes within a literature (Rubin, 2004), while evaluating annual citation rates may indicate how the science is trending (Lipsman and Lozano, 2012).

The primary objective of our review was to identify and characterize TBI publications that have (1) the greatest number of lifetime citations and (2) the highest annual citation rates. We categorized and analyzed top-cited articles according to characteristics such as their primary focus, sample, design, and country of correspondence. Other groups have conducted similar analyses within different areas of research (Yang and Pan, 2006; Lefaivre et al., 2010; Ponce and Lozano, 2010; Shadgan et al., 2010; Ibrahim et al., 2012; Lipsman and Lozano, 2012; Lipsman et al., 2014). Collectively, the publications reviewed here highlight some of the most influential articles in TBI, and also the studies that may be shaping the direction in which this growing field is heading.

## Methods

We used the publically available software Harzing’s Publish or Perish v.4.6.3 (Harzing, 2007) to conduct our citation analysis. This software computes various citation metrics after collecting raw citation data through Google Scholar. It should be noted that other groups have conducted bibliometric citation analyses using Harzing’s Publish or Perish (Lipsman and Lozano, 2012; Lipsman et al., 2014). Citation metrics are accurate to May 1, 2014.

Our search included a comprehensive set of terms. In particular, we searched for articles that contained the term “head trauma,” “head injury,” “head injuries,” “head injured,” “brain trauma,” “brain injury,” “brain injuries,” “brain injured,” “traumatic brain injury,” “traumatic brain injuries,” “TBI,” “concussion,” or “concussions” in the study title. We manually reviewed the full list of the top-cited TBI articles generated by Harzing’s Publish or Perish (Harzing, 2007) in consecutive order until a total of 50 articles that met our inclusion and exclusion criteria (outlined below) were identified. We also sorted the data in Harzing’s Publish or Perish (Harzing, 2007) by “citations/year” to identify the 50 TBI articles, meeting our inclusion and exclusion criteria, with the highest annual citation rates. There were no restrictions on date of publication.

**Figure 1:**
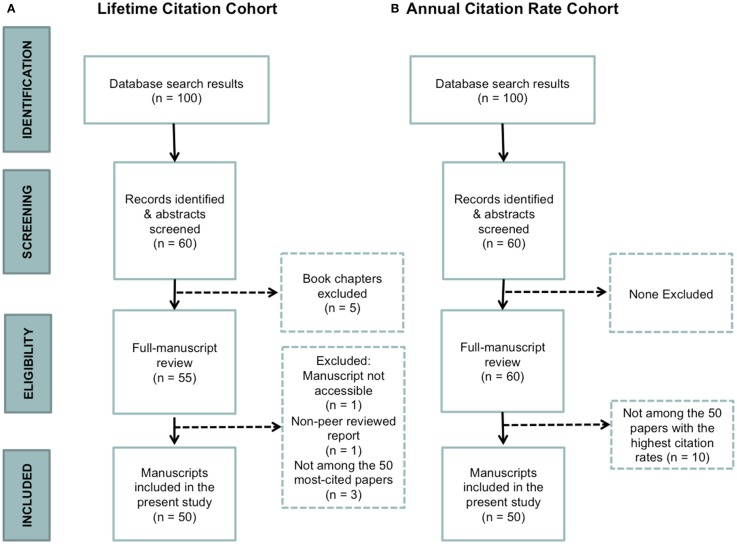
**Flow diagram representing the study selection process**. **(A)** Lifetime citation cohort. **(B)** Annual citation rate cohort.

To be included in our final sample, it was necessary for TBI to be the primary focus of the article (e.g., the article could not be about acquired brain injuries in general), the full article to be electronically accessible, and for the article to be peer reviewed. We included primary publications, consensus statements/guidelines, commentaries, and review articles. An article was excluded if it was not peer reviewed, and/or if it was a report or book chapter (Figure 1).

A grounded theory methodology (Strauss and Corbin, 1990) was used to code each article, with respect to overall focus/topic. After independently coding the same set of 40 articles (at which point data saturation was achieved), the authors met to reach a consensus on the categorization strategy and ensure that similar codes were being grouped together consistently. After coding articles from both citation cohorts (i.e., the 50 most-cited articles in TBI and the 50 articles with the highest annual citation rates), differences in coding were reconciled by debate until consensus was reached. Both cohorts of articles were analyzed similarly. Only descriptive statistics were performed.

## Results

The publications that comprise the two citation cohorts are listed in Tables 1 and 2. On average, the 50 most-cited articles in TBI accumulated a lifetime total of 834.8 ± 248. 8 citations (range: 574–1645). The average annual citation rate of these papers was 48.0 ± 43.4 citations per year, with a range between 8.1 and 192.7. In contrast, the annual citation rate of the 50 TBI papers that have accumulated the most citations per year since publication was 65.5 ± 35.4 citations per year (range: 39.1–192.7). The absolute number of citations this latter cohort of papers accumulated since publication ranged from 169 to 1645, with an average of 654.7 ± 350.6 lifetime citations. Over half (27/50, 54.0%) of the publications with the highest annual citation rates were common to the 50 TBI papers with the most lifetime citations.

**Table 1:** **TBI publications with the most lifetime citations (*n* = 50)**.

Rank	First author	Study title	Year of publication	Number of citations	Annual citation rate (citations/year)	Topic/focus
1	Chestnut, RM	The role of secondary brain injury in determining outcome from severe head injury	1993	1645	78.3	Predictor of outcome
2	Gronwall, DM	Paced auditory serial-addition task: a measure of recovery from concussion	1977	1485	40.1	Assessment measure
3	Brines, ML	Erythropoietin crosses the blood–brain barrier to protect against experimental brain injury	2000	1318	94.1	Treatment
4	Faden, A	The role of excitatory amino acids and NMDA receptors in traumatic brain injury	1989	1300	52.0	Treatment
5	Marion, DW	Treatment of traumatic brain injury with moderate hypothermia	1997	1215	71.5	Treatment
6	Clifton, GL	Lack of effect of induction of hypothermia after acute brain injury	2001	1182	90.9	Treatment
7	Hoge, CW	Mild traumatic brain injury in soldiers returning from Iraq	2008	1156	192.7	Predictor of outcome
8	Davalos, D	ATP mediates rapid microglial response to local brain injury *in vivo*	2005	1081	120.1	Pathology/natural history
9	Rimel, RW	Disability caused by minor head injury	1981	1023	31.0	Predictor of outcome
10	Adams, JH	Diffuse axonal injury due to non-missile head injury in human beings: an analysis of 45 cases	1982	1010	31.6	Pathology/natural history
11	Muizelaar, JP	Adverse effects of prolonged hyperventilation in patients with severe head injury: a randomized clinical trial	1991	987	42.9	Treatment
12	Thurman, DJ	Traumatic brain injury in the United Stats: a public health perspective	1999	963	64.2	Epidemiology
13	Becker, DP	The outcome from severe head injury with early diagnosis and intensive management	1977	941	25.4	Treatment
14	Langlois, JA	The epidemiology and impact of traumatic brain injury: a brief overview	2006	930	116.3	Epidemiology
15	McCrory, P	Consensus statement on concussion in sport: The 3rd International Conference on Concussion in Sport held in Zurich	2009	907	181.4	Guidelines and consensus statements
16	Marshall, LF	A new classification of head injury based on computed tomography	1991	899	39.1	Pathology/natural history
17	Marmarou, A	A new model of diffuse brain injury in rats: part 1: pathophysiology and biomechanics	1994	887	44.4	Experimental model of TBI
18	Ommaya, AK	Cerebral concussion and traumatic unconsciousness: correlation of experimental and clinical observations on blunt head injuries	1974	811	20.3	Experimental model of TBI
19	Adams, JH	Diffuse axonal injury in head injury: definition, diagnosis, and grading	1989	810	32.4	Pathology/natural history
20	Obrist, WD	Cerebral blood flow and metabolism in comatose patients with acute head injury: relationship to intracranial hypertension	1984	800	26.7	Predictor of outcome
21	McIntosh, TK	Traumatic brain injury in the rat: characterization of a lateral fluid-percussion model	1989	797	31.9	Predictor of outcome
22	Jennett, B	Disability after severe head injury: observations on the use of the Glasgow Outcome Scale	1981	781	23.7	Predictor of outcome
23	Dixon, CE	A fluid-percussion model of experimental brain injury in the rat	1987	764	28.3	Predictor of outcome
24	Miller, JD	Significance of intracranial hypertension in severe head injury	1977	761	20.6	Predictor of outcome
25	Alexander, MP	Mild traumatic brain injury: pathophysiology, natural history, and clinical management	1995	759	40.0	Pathology/natural history
26	Levin, HS	Neurobehavioral outcome following minor head injury: a three-center study	1987	756	28.0	Predictor of outcome
27	Katayama, Y	Massive increases in extracellular potassium and the indiscriminate release of glutamate following concussive brain injury	1990	754	31.4	Pathology/natural history
28	Levin, HS	The Galveston Orientation and Amnesia Test: a practical scale to assess cognition after head injury	1979	752	21.5	Assessment measure
29	Bullock, R	Guidelines for the management of severe head injury	1996	740	41.1	Guidelines and consensus statements
30	Dixon, CE	A controlled cortical impact model of traumatic brain injury in the rat	1991	737	32.0	Predictor of outcome
31	Robertson, IH	Oops!: performance correlates of everyday attentional failures in traumatic brain injured and normal subjects	1997	714	42.0	Assessment measure
32	Guskiewicz, KM	Cumulative effects associated with recurrent concussion in collegiate football players: The NCAA Concussion Study	2003	670	60.9	Epidemiology
33	Bouma, GJ	Cerebral circulation and metabolism after severe traumatic brain injury: the elusive role of ischemia	1991	665	28.9	Predictor of outcome
34	Aubry, M	Summary and agreement statement of The first International Conference on Concussion in Sport, Vienna 2001	2002	640	53.3	Guidelines and consensus statements
35	Marmarou, A	Impact of ICP instability and hypertension on outcome in patients with severe head trauma	1991	640	27.8	Predictor of outcome
36	Stiell, IG	The Canadian CT Head Rule for patients with minor head injury	2001	637	49.0	Guidelines and consensus statements
37	McCrory, P	Summary and agreement statement of The 2nd International Conference on Concussion in Sport, Prague 2004	2005	629	69.9	Guidelines and consensus statements
38	Gronwall, DM	Delayed recovery of intellectual function after minor head injury	1974	625	15.8	Predictor of outcome
39	Miller, JD	Further experience in the management of severe head injury	1981	624	18.9	Predictor of outcome
40	Strich, SJ	Diffuse degeneration of the cerebral white matter in severe dementia following head injury	1956	606	10.5	Pathology/natural history
41	Yakolev, AG	Activation of CPP32-like caspases contributes to neuronal apoptosis and neurological dysfunction after traumatic brain injury	1997	603	35.5	Pathology/natural history
42	Jennett, B	Predicting outcome in individual patients after severe head injury	1976	600	15.8	Predictor of outcome
43	Collins, MW	Relationship between concussion and neuropsychological performance in college football players	1999	594	39.6	Predictor of outcome
44	Clifton, GL	A phase II study of moderate hypothermia in severe brain injury	1993	591	28.1	Treatment
45	Brooks, N	The 5-year outcome of severe blunt head injury: a relative’s view	1986	589	21.0	Pathology/natural history
46	Denny-Brown, D	Experimental cerebral concussion	1941	588	8.1	Experimental model of TBI
47	Shiozaki, T	Effect of mild hypothermia on uncontrollable intracranial hypertension after severe head injury	1993	585	27.9	Treatment
48	Narayan, RK	Clinical trials in head injury	2002	580	48.3	Guidelines and consensus statements
49	Teasdale, GM	Association of apolipoprotein E polymorphism with outcome after head injury	1997	574	33.6	Predictor of outcome
50	Rink, A	Evidence of apoptotic cell death after experimental traumatic brain injury in the rat	1995	574	30.1	Pathology/natural history

**Table 2:** **TBI publications with the highest annual citation rates (*n* = 50)**.

Rank	First author	Study title	Year of publication	Number of citations	Annual citation rate (citations/year)	Topic/focus
1	Hoge, CW	Mild traumatic brain injury in soldiers returning from Iraq	2008	1156	192.7	Predictor of outcome
2	McCrory, P	Consensus statement on concussion in sport: The 3rd International Conference on Concussion in Sport held in Zurich	2009	907	181.4	Guidelines and consensus statements
3	McCrory, P	Consensus statement on concussion in sport: The 4th International Conference on Concussion in Sport held in Zurich, November, 2012	2013	169	169.0	Guidelines and consensus statements
4	Davalos, D	ATP mediates rapid microglial response to local brain injury *in vivo*	2005	1081	120.1	Pathology/natural history
5	Langlois, JA	The epidemiology and impact of traumatic brain injury: a brief overview	2006	930	116.3	Epidemiology
6	Cooper, DJ	Decompressive craniectomy in diffuse traumatic brain injury	2011	320	106.7	Treatment
7	McKee, AC	Chronic traumatic encephalopathy in athletes: progressive tauopathy following repetitive head injury	2009	510	102.0	Predictor of outcome
8	Brines, ML	Erythropoietin crosses the blood–brain barrier to protect against experimental brain injury	2000	1318	94.1	Treatment
9	Clifton, GL	Lack of effect of induction of hypothermia after acute brain injury	2001	1182	90.9	Treatment
10	Chestnut, RM	The role of secondary brain injury in determining outcome from severe head injury	1993	1645	78.3	Predictor of outcome
11	Maas, AIR	Moderate and severe traumatic brain injury in adults	2008	458	76.3	Epidemiology
12	Marion, DW	Treatment of traumatic brain injury with moderate hypothermia	1997	1215	71.5	Treatment
13	McCrory, P	Summary and agreement statement of The 2nd International Conference on Concussion in Sport, Prague 2004	2005	629	69.9	Guidelines and consensus statements
14	Thurman, DJ	Traumatic brain injury in the United Stats: a public health perspective	1999	963	64.2	Epidemiology
15	Warden, D	Military TBI during the Iraq and Afghanistan wars	2006	490	61.3	Epidemiology
16	Guskiewicz, KM	Cumulative effects associated with recurrent concussion in collegiate football players: The NCAA Concussion Study	2003	670	60.9	Epidemiology
17	Kraus, MF	White matter integrity and cognition in chronic traumatic brain injury: a diffusion tensor imaging study	2007	426	60.9	Pathology/natural history
18	Tagliaferri, F	A systematic review of brain injury epidemiology in Europe	2006	485	60.6	Epidemiology
19	Kuppermann, N	Identification of children at very low risk of clinically important brain injuries after head trauma: a prospective cohort study	2009	303	60.4	Predictor of outcome
20	Okie, S	Traumatic brain injury in the war zone	2005	512	56.9	Predictor of outcome
21	Aubry, M	Summary and agreement statement of The first International Conference on Concussion in Sport, Vienna 2001	2002	640	53.3	Guidelines and consensus statements
22	Saatman, KE	Classification of traumatic brain injury for targeted therapies	2008	320	53.3	Pathology/natural history
23	Roberts	Effect of intravenous corticosteroids on death within 14 days in 10,008 adults with clinically significant head injury (MRC CRASH trial): randomized placebo-controlled trial	2004	533	53.3	Treatment
24	Schiff, ND	Behavioral improvements with thalamic stimulation after severe traumatic brain injury	2007	371	53.0	Treatment
25	Faden, A	The role of excitatory amino acids and NMDA receptors in traumatic brain injury	1989	1300	52.0	Treatment
26	Carroll, L	Prognosis for mild traumatic brain injury: results of the WHO collaborating center task force on mild traumatic brain injury	2004	519	51.9	Pathology/natural history
27	McCrea, M	Acute effects and recovery time following concussion in collegiate football players: The NCAA Concussion Study	2003	566	51.5	Pathology/natural history
28	Aarabi, B	Outcome following decompressive craniectomy for malignant swelling due to severe head injury	2006	410	51.3	Treatment
29	Schneiderman, AI	Understanding sequelae of injury mechanisms and mild traumatic brain injury incurred during the conflicts in Iraq and Afghanistan: persistent post-concussive symptoms and post-traumatic stress disorder	2008	307	51.2	Epidemiology
30	Werner, C	Pathophysiology of traumatic brain injury	2007	348	49.7	Pathology/natural history
31	Stiell, IG	The Canadian CT Head Rule for patients with minor head injury	2001	637	49.0	Guidelines and consensus statements
32	Bullock, MR	Guidelines for the management of severe traumatic brain injury. Editor’s commentary	2007	343	49.0	Guidelines and consensus statements
33	Bruns, J	The epidemiology of traumatic brain injury: a review	2003	536	48.7	Epidemiology
34	Narayan, RK	Clinical trials in head injury	2002	580	48.3	Guidelines and consensus statements
35	Hutchison, JS	Hypothermia therapy after traumatic brain injury in children	2008	287	47.7	Treatment
36	Halstead, ME	Sport-related concussion in children and adolescents	2010	190	47.5	Guidelines and consensus statements
37	MRCCT Collaborators	Predicting outcome after traumatic brain injury: practical prognostic models based on large cohort of international patients	2008	284	47.3	Predictor of outcome
38	Wright, DW	ProTECT: a randomized clinical trial of progesterone for acute traumatic brain injury	2007	323	46.1	Treatment
39	Rutland-Brown, W	Incidence of traumatic brain injury in the United States, 2003	2006	362	45.3	Epidemiology
40	Guskiewicz, KM	Association between recurrent concussion and late-life cognitive impairment in retired professional football players	2005	402	44.7	Predictor of outcome
41	Marmarou, A	A new model of diffuse brain injury in rats: part 1: pathophysiology and biomechanics	1994	887	44.4	Experimental model of TBI
42	Muizelaar, JP	Adverse effects of prolonged hyperventilation in patients with severe head injury: a randomized clinical trial	1991	987	42.9	Treatment
43	Robertson, IH	Oops!: performance correlates of everyday attentional failures in traumatic brain injured and normal subjects	1997	714	42.0	Assessment measure
44	Cassidy, JD	Incidence, risk factors and prevention of mild traumatic brain injury: results of the WHO Collaborating Centre Task Force on Mild Traumatic Brain Injury	2004	420	42.0	Epidemiology
45	Panikashvili, D	An endogenous cannabinoid (2-AG) is neuroprotective after brain injury	2001	536	41.2	Pathology/natural history
46	Bullock, R	Guidelines for the management of severe head injury	1996	740	41.1	Guidelines and consensus statements
47	Gronwall, DM	Paced auditory serial-addition task: a measure of recovery from concussion	1977	1485	40.1	Assessment measure
48	Alexander, MP	Mild traumatic brain injury: pathophysiology, natural history, and clinical management	1995	759	40.0	Pathology/natural history
49	Collins, MW	Relationship between concussion and neuropsychological performance in college football players	1999	594	39.6	Predictor of outcome
50	Marshall, LF	A new classification of head injury based on computed tomography	1991	899	39.1	Pathology/natural history

**Figure 2:**
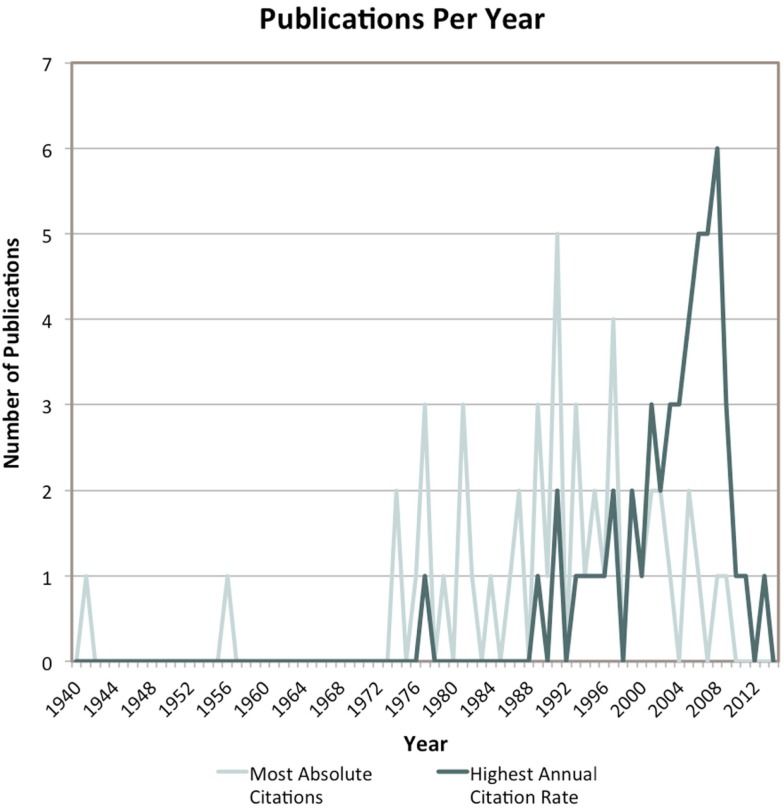
**Publication year of each of the articles that comprise the two citation cohorts**.

Most papers with the greatest number of lifetime citations were published over a 30-year span starting in the mid-1970s (mean year of publication: 1990 ± 14.9 years). Conversely, the majority of TBI-specific papers with the highest annual citation rates were published within the last 15 years (mean year of publication: 2003 ± 6.7 years) (Figure 2). Both citation cohorts are further characterized in Table 3.

**Table 3:** **Characterization of the two citation cohorts**.

		TBI publications with the most lifetime citations: *n* (%)	TBI publications with the highest annual citation rates: *n* (%)
Severity	Mild	15 (30.0)	18 (36.0)
	Mild-to-moderate	1 (2.0)	0 (0.0)
	Mild-to-severe	9 (18.0)	14 (28.0)
	Moderate	0 (0.0)	0 (0.0)
	Moderate-to-severe	2 (4.0)	3 (6.0)
	Severe	19 (38.0)	11 (22.0)
	Unspecified	4 (8.0)	4 (8.0)
Sample	Animal	12 (24.0)	5 (10.0)
	Human	38 (76.0)	45 (90.0)
	Pediatric	3 (7.9)	4 (8.9)
	Sport	9 (23.7)	9 (20.0)
	Veteran	3 (7.9)	4 (8.9)
Design	Case-series	3 (6.0)	1 (2.0)
	Commentary	0 (0.0)	1 (2.0)
	Consensus statement/guidelines	4 (8.0)	6 (12.0)
	Cross-sectional	0 (0.0)	2 (4.0)
	Prospective cohort; uncontrolled	14 (28.0)	8 (16.0)
	Prospective cohort; controlled	6 (12.0)	4 (8.0)
	Randomized control trial	5 (10.0)	7 (14.0)
	Retrospective cohort	2 (4.0)	2 (4.0)
	Review	6 (12.0)	14 (28.0)
	Unspecified	10 (20.0)	5 (10.0)
Country of correspondence	Australia	2 (4.0)	4 (8.0)
	Belgium	0 (0.0)	1 (2.0)
	Canada	2 (4.0)	5 (10.0)
	Germany	0 (0.0)	1 (2.0)
	Israel	0 (0.0)	1 (2.0)
	Japan	1 (2.0)	0 (0.0)
	New Zealand	2 (4.0)	1 (2.0)
	United Kingdom	9 (18.0)	2 (4.0)
	United States of America	34 (68.0)	33 (66.0)
	Multi-national	0 (0.0)	2 (4.0)
Product of international collaboration		8 (16.0)	14 (28.0)
Average number of authors		6.5 ± 6.2	10.3 ± 10.6

All papers were classified into one of seven general categories. Table 4 provides the distribution of papers from both citation cohorts into these seven categories (and sub-categories where appropriate). A more detailed discussion of category-specific findings follows.

**Table 4:** **Categorical dissection of publications in both citation cohorts**.

	Publications with the most lifetime citations: *n* (%)	Publications with the highest annual citation rates: *n* (%)
Predictor of outcome	17 (34.0)	8 (16.0)
Cognitive	3 (17.6)	2 (25.0)
Gross	5 (29.4)	3 (37.5)
Mortality	5 (29.4)	1 (12.5)
Neurological	4 (23.5)	2 (25.0)
Pathology/natural history	10 (20.0)	9 (18.0)
Gross	2 (20.0)	1 (11.1)
Functional	1 (10.0)	3 (33.3)
Histological	4 (40.0)	2 (22.2)
Physiological	3 (30.0)	3 (33.3)
Treatment	8 (16.0)	11 (22.0)
Pharmacological intervention	2 (25.0)	4 (36.4)
Hyperventilation	1 (12.5)	1 (9.1)
Hypothermia	4 (50.0)	3 (27.3)
Neurosurgical	1 (12.5)	3 (27.3)
Guidelines and consensus statements	6 (12.0)	9 (18.0)
Epidemiology	3 (6.0)	10 (20.0)
Assessment measure	3 (6.0)	2 (4.0)
Experimental model of TBI	3 (6.0)	1 (2.0)

*Note: The “*Predictor of outcome*” category was specific to articles investigating the relationship between a variable and outcome at a later time point, whereas the “*Pathology/natural history*” category was appropriated to studies investigating the natural course and manifestations of TBI*.

### Predictor of outcome

Of the 50 most frequently cited articles in TBI, 17 (17/50, 34.0%) primarily studied predictors of outcome. Most predictor studies involved human subjects (14/17, 82.4%), with a subset focusing exclusively on athletes (1/14, 7.1%) and war veterans (1/14, 7.1%). Nearly half of all predictor articles (8/17, 47.1%) studied severe TBI, while five centered on mild TBI (5/17, 29.4%); the remainder (4/17, 23.5%) were composed of a heterogeneous sample with respect to injury severity. None of the top-cited studies investigating predictors of outcome were randomized control trials, although many were prospectively conducted (15/17, 88.2%).

Comparatively, of the 50 articles with the highest annual citation rates, only eight (8/50, 16.0%) investigated predictors of outcome. Of these eight studies, half (4/8, 50.0%) were common to the 50 top-cited articles in TBI. Most predictor studies in the citation rate cohort were prospectively conducted (4/8, 50.0%), and half of all predictor studies (4/8, 50.0%) focused on a specialty population (i.e., athletes or war veterans). However, in comparison to the cohort of papers with the most lifetime citations, a greater percentage of predictor studies with the highest annual citation rates were specific to mild TBI (3/8, 37.5%).

### Pathology/natural history

The 50 top-cited articles in TBI included 10 studies (10/50, 20.0%) primarily focused on characterizing pathological outcome following brain injury. Nine of the pathology papers (9/10, 90.0%) were primary research articles; the other was a review article (1/10, 10.0%). The majority of pathology articles were of a prospective design (8/10, 80.0%) and involved human subjects (6/10, 60%). Severe TBI was studied in four articles (4/10, 40.0%), mild TBI in three papers (3/10, 30.0%), and two studies (2/10, 20.0%) had a mixed sample with respect to TBI severity. Information on TBI severity was not available for one study (1/10, 10.0%).

Of the 50 articles with the highest annual citation rates, nine (9/50, 18.0%) focused on pathology/natural history; six of these were common to the 50 top-cited articles in TBI. Relative to the cohort of papers with the most absolute citations, a greater proportion of the citation rate studies were review articles (4/9, 44.4%) and involved human subjects (7/9, 77.8%). A dissection by TBI severity revealed that mild TBI was studied in three papers (3/9, 33.3%), severe TBI in another (1/9, 11.1%), while samples with heterogeneous injury severity were studied in three articles (3/9, 33.3%). TBI severity could not be determined in the remaining papers (2/9, 22.2%).

### Treatment

Treatment was the focus of eight of the 50 top-cited articles in TBI (8/50, 16.0%). The majority of these articles (6/8, 75.0%) involved human subjects; the remainder studied pre-clinical treatment options for TBI in animal models (2/8, 25.0%). Most articles studied treatment in the context of severe TBI (6/8, 75.0%), and many treatment articles were randomized control trials (5/8, 62.5%).

Of the 50 TBI articles with the highest annual citation rates, the subject of 11 (11/50, 22.0%) was treatment. When compared to the cohort of treatment studies with the most lifetime citations, a similar proportion of these articles involved human subjects (9/11, 81.8%) and focused on moderate-to-severe or severe TBI (9/11, 81.8%). TBI treatment studies with the highest annual citation rates, however, studied interventions, such as administration of corticosteroids (1/11, 9.1%) or progesterone (1/11, 9.1%), which the top-cited treatment articles did not. Relative to papers with the most lifetime citations, a similar percentage of papers in the citation rate cohort (7/11, 63.6%) were randomized controlled trials.

### Guidelines and consensus statements

Six of the 50 top-cited TBI articles (6/50, 12.0%) were guidelines and/or consensus statements. Two-thirds (4/6, 66.7%) of these articles were a product of international collaboration. The majority of these articles (4/6, 66.7%) pertained to mild TBI, including three (3/6, 50.0%) focusing exclusively on sports-related concussion. One consensus statement (1/6, 16.7%) concerned severe TBI, and another (1/6, 16.7%) provided guidelines on how to manage TBI of all severities.

Of the 50 TBI articles with the highest annual citation rates, nine (9/50, 18.0%) were guidelines and consensus statements; six of these were common to the 50 most-cited articles in TBI. An analysis by TBI severity revealed that mild TBI was the focus of six articles (6/9, 66.7%), three of which (3/6, 50.0%) were exclusively focused on concussion. One consensus statement (1/9, 11.1%) discussed pediatric head injuries specifically.

### Epidemiology

The 50 most-cited articles in TBI included three (3/50, 6.0%) epidemiological studies. One of these articles (1/3, 33.3%) studied the full spectrum of brain injury (e.g., mild-to-severe), while another (1/3, 33.3%) provided an epidemiological context to mild TBI only. The latter study was specific to TBI in sport.

In contrast, of the 50 TBI articles with the highest annual citation rates, 10 (10/50, 20.0%) were epidemiological. Half (5/10, 50.0%) of these articles studied the full spectrum of TBI severity, with one study (1/10, 10.0%) focusing exclusively on mild TBI. The single article studying the epidemiology of mild TBI did so in the context of sports. Another article (1/10, 10.0%) investigated the epidemiology of all TBI severities in war veterans.

### Assessment measures

Of the 50 top-cited articles in TBI, three (3/50, 6.0%) primarily focused on assessment measures. All three studies (3/3, 100.0%) were prospective cohort investigations of cognitive assessment measures for information processing speed, sustained attention, or post-trauma orientation and amnesia. Two of these articles (2/3, 66.7%) focused on mild TBI and the third (1/3, 33.3%) explored assessment measures for TBI of any severity. The two studies specific to mild TBI in the most lifetime citation cohort were also among the 50 articles with the highest annual citation rates.

### Experimental model of TBI

Three papers (3/50, 6.0%) describing an experimental model of TBI (e.g., fluid-percussion models) were identified in the cohort of 50 most-cited TBI articles; one (1/3, 33.3%) of these was common to the citation rate cohort. All three articles were animal studies. One study (1/3, 33.3%) developed a model to explore mild TBI, whereas the others (2/3, 66.7%), which were also common to the citation rate cohort, centered on a model that permitted investigation of mild-to-severe TBI in animals.

## Discussion

In comparing TBI papers with the most lifetime citations to those with the highest annual citation rates, it is possible to gage, respectively, which papers have had the greatest influence in TBI, and which articles are currently discussed, referenced, and shaping the field. Below, we provide a general discussion of our findings, which is followed by a category-specific commentary where appropriate.

Our data show that studies on mild TBI are currently accumulating the most citations per year, although studies on severe TBI have been more widely cited (Table 3). This suggests that mild TBI is presently a central point of discussion in the field of brain injury, perhaps because mild TBI is the most prevalent form of TBI (Faul et al., 2010) and has, as a result, the most scope for preventive research; is of social and public concern, given its incidence in sports and the military (Langlois et al., 2006; Chen and D’Esposito, 2010; Cusimano et al., 2013); is a risk factor for other disorders (e.g., depression and anxiety) and neurodegenerative diseases such as chronic traumatic encephalopathy and Alzheimer’s disease (Plassman et al., 2000; McCauley et al., 2001; Holsinger et al., 2002; Fleminger et al., 2003; Seel et al., 2003; Gavett et al., 2010, 2011; Masel and DeWitt, 2010; Stern et al., 2011), and thereby draws interest and citations across a number of neuroscience disciplines; may now be extensively researched to overcome some of the documented shortcomings of former studies on mild TBI (Carroll et al., 2004). Furthermore, relative to papers with the most lifetime citations, a greater proportion of articles that are accumulating the most citations annually involved human subjects (Table 3). This suggests a shifted focus from animal to clinical research, and perhaps a piqued interest in understanding, in particular, human brain and behavior after TBI. In addition, studies with the highest annual citation rates involved, on average, more routine international collaboration than papers with the most lifetime citations (Table 3). Increased international collaboration may ultimately benefit our understanding of TBI by facilitating comparative effectiveness treatment research, the development of brain injury biomarkers, and an improved ability to predict outcome following TBI (Tosetti et al., 2013). Moreover, the articles that comprised the citation rate cohort were, on average, authored by a greater number of individuals (Table 3). As larger groups conducted these studies, they may, therefore, benefit from greater scientific diversity and perspective.

Speculating on future trends in TBI research would suggest a sustained focus on treatment of TBI, given that patients continue to experience persistent cognitive and emotional impairment more than 5 years following mild trauma (Konrad et al., 2011). It may also be reasonable to expect an increase in research activity on assessment measures for TBI, given the push to be able to more reliability and immediately identify brain injury in the context of sports (Charleswell et al., 2014). As TBI research evolves and new knowledge is assimilated into current understanding and practice, is also likely that there will be greater research activity surrounding guidelines and consensus statements.

We would like to note that increased research activity in one subfield may influence activity in another. For example, a shifted research focus on treatment of TBI may engender downstream epidemiological research designed to assess treatment effects at the population level. Likewise, an increased focus on treatment may result in updated guidelines or consensus statements. Below, however, only category-specific findings are discussed.

### Predictor of outcome

Over a third of the 50 top-cited articles in TBI studied predictors of outcome, indicating that this subfield of brain injury has been extensively researched. Moreover, eight of the 50 articles with the highest yearly citation rates focused on predictors of outcome, and half of these were also among the 50 top-cited articles in TBI. This indicates that novel research on predictors of outcome – not only former, seminal work in this subfield – continues to be discussed and cited widely. A sustained research effort into predictors of outcome is required to inform clinicians as to which patients are at greatest risk of poor long-term outcome, and, therefore, should be targeted for a particular management strategy or therapeutic intervention. Moreover, articles with the highest annual citation rates most often predicted cognitive, gross, and neurological outcomes following TBI, and not mortality, like many of 50 most-cited publications in TBI. A reduced emphasis on predicting mortality following brain injury may be commensurate with our improved ability to save the lives of TBI patients through primary prevention (e.g., seatbelt use) and case management (Stiefel et al., 2005).

### Pathology/natural history

Investigations into pathological outcome and natural history following TBI are important for understanding the recovery and progression of brain injury. The most highly cited pathology/natural history studies examined histological outcomes and physiological response to trauma; however, the articles with the highest citation rates more often investigated functional outcomes post-TBI. This suggests a shifted focus, wherein patient-centered functional outcomes have become a more central point of research. This echoes the growing appreciation for the functional sequelae of brain injuries (Morton and Wehman, 1995), and identification of barriers to functional recovery that prevent restoration of pre-morbid abilities (Powell et al., 2001).

### Treatment

Two treatment articles with the highest annual citation rates were not common to the 50 most-cited articles in TBI. This suggests that some TBI treatment articles, despite not yet accumulating the requisite number of citations to be among the most-cited articles in TBI, are currently being regularly discussed and referenced within the scientific community. Discussion of and research into new treatment options for TBI is necessary given the limited efficacy of many currently available brain injury treatment and management options (Maas et al., 2012; Ponsford et al., 2014a,b; Velikonja et al., 2014), despite our increased pathophysiological understanding of TBI (Zitnay et al., 2008).

### Guidelines and consensus statements

The greatest number of guidelines and consensus statements specific to TBI were identified in the citation rate cohort, indicating a current increase in scientific discussion on this topic. However, it should be noted that recent research indicates that, on average, TBI guidelines are based on low levels of evidence (Maas et al., 2012). This highlights the need to design and conduct studies on TBI that can provide high-level evidence that will advance the science and inform TBI guidelines of a higher standard. Nonetheless, the guidelines and consensus statements that are currently being referenced are particular to mild TBI and concussion. The observed increase in referencing of concussion and mild TBI guidelines echoes the growing interest and research activity in this field (Table 3).

### Epidemiology

We observed that a greater proportion of the citation rate cohort comprised epidemiological studies than the absolute citation cohort. This suggests that our epidemiological knowledge of TBI is continually developing, re-contextualizing our understanding of the prevalence of TBI and the associated scope for prevention, characteristics of vulnerable populations, and how to distribute resources to manage TBI. Continued epidemiological research into TBI is required given that the incidence of this injury is changing (Maas et al., 2012). This is largely due to increased availability and use of motor vehicles in developing nations, and, therefore, greater potential for TBIs to be caused by motor vehicle collisions in these countries (Maas et al., 2008). In developed nations, moreover, rates of TBI are also on the rise in the elderly (Faul et al., 2010), potentially due to fall-related brain injuries. Given the above, the epidemiology of TBI will continue to change and require ongoing investigation. Improving and updating our understanding of the epidemiology of TBI provides the backdrop necessary for more downstream lines of research, which require an understanding of the scale of brain injury, such as prevention, treatment, and management.

## Limitations

Our review, similar to other citation studies, is subject to a number of limitations. Chief among these is the possibility that our search terms, albeit comprehensive, did not permit identification of every one of the top-cited papers in TBI. However, because we used Harzing’s Publish or Perish (Harzing, 2007) for our analysis, our search was biased toward inclusivity. This is because Harzing’s Publish or Perish (Harzing, 2007) collects raw citation data through Google Scholar, which is more inclusive in terms of which journals it indexes than other search engines such as the Institute for Scientific Information (ISI) Web of Science, which indexes only ISI journals (Lipsman and Lozano, 2011). Furthermore, the citation metrics that were computed in the present review have likely changed since we completed our analyses, given that the TBI literature is being continually cited. Any recent citations of the papers included in the present review may alter the ranking of some of the top-cited articles in TBI, though the overall content of the two citation cohorts is unlikely to change substantially in short intervals of time.

Furthermore, recent years have seen a growth in the number of scientific publications related to TBI. A search of the Medical Subject Headings (MeSH) keywords “traumatic brain injury,” “traumatic brain injuries,” “concussion,” and “concussions” in PubMed shows a stable growth in the number of TBI-related publications since 2004. In particular, between 2004 and 2013, 1733 TBI-specific papers were published, more than twice the 833 papers on brain injury published from 1994-2003. Therefore, as the citation rates we present do not control for general growth in the TBI field, our findings should be interpreted with caution.

There are also other measures of citation impact, including the h-index, g-index, and e-index. Although these metrics have unique value (Zhang, 2009, 2010), we did not include them in the present review, as we were not investigating citations by author or across specialties, institutions, or countries. We also did not control for the effects of self-citation or differences in citation practices across medical specialties (Kulkarni et al., 2007). Our review is also cross-sectional, and did not permit a longitudinal investigation. Future citation studies using alternative citation metrics and/or evaluating citation trends over time may be of additional value.

## Conclusion

The present review provides a cross-sectional summary of some of the most influential studies in TBI, highlighting areas of research that require further investigation and development. Although studies on severe brain injury and predictors of outcome following TBI have been cited most extensively, the current research focus appears to be on mild TBI and investigating treatment strategies for brain injury. Our review is not designed to supplant systematic reviews or meta-analyses in TBI, but rather synthesize the literature uniquely to permit a novel analysis of TBI research. As the TBI literature evolves, it will be important for future citation studies to re-evaluate existing patterns and trends within this growing field of research.

## Conflict of Interest Statement

The authors declare that the research was conducted in the absence of any commercial or financial relationships that could be construed as a potential conflict of interest.
